# Detecting and Analyzing Suicidal Ideation on Social Media Using Deep Learning and Machine Learning Models

**DOI:** 10.3390/ijerph191912635

**Published:** 2022-10-03

**Authors:** Theyazn H. H. Aldhyani, Saleh Nagi Alsubari, Ali Saleh Alshebami, Hasan Alkahtani, Zeyad A. T. Ahmed

**Affiliations:** 1 Applied College in Abqaiq, King Faisal University, P.O. Box 400, Al-Ahsa 31982, Saudi Arabia; aalshebami@kfu.edu.sa; 2 Department of Computer Science, Dr. Babasaheb Ambedkar Marathwada University, Aurangabad 431004, India; salehalsubri2018@gmail.com (S.N.A.); zeyad.ahmed2019@yahoo.com (Z.A.T.A.); 3 College of Computer Science and Information Technology, King Faisal University, P.O. Box 400, Al-Ahsa 31982, Saudi Arabia; hsalkahtani@kfu.edu.sa

**Keywords:** machine learning, artificial intelligence, suicidal ideation, LIWC-22

## Abstract

Individuals who suffer from suicidal ideation frequently express their views and ideas on social media. Thus, several studies found that people who are contemplating suicide can be identified by analyzing social media posts. However, finding and comprehending patterns of suicidal ideation represent a challenging task. Therefore, it is essential to develop a machine learning system for automated early detection of suicidal ideation or any abrupt changes in a user’s behavior by analyzing his or her posts on social media. In this paper, we propose a methodology based on experimental research for building a suicidal ideation detection system using publicly available Reddit datasets, word-embedding approaches, such as TF-IDF and Word2Vec, for text representation, and hybrid deep learning and machine learning algorithms for classification. A convolutional neural network and Bidirectional long short-term memory (CNN–BiLSTM) model and the machine learning XGBoost model were used to classify social posts as suicidal or non-suicidal using textual and LIWC-22-based features by conducting two experiments. To assess the models’ performance, we used the standard metrics of accuracy, precision, recall, and F1-scores. A comparison of the test results showed that when using textual features, the CNN–BiLSTM model outperformed the XGBoost model, achieving 95% suicidal ideation detection accuracy, compared with the latter’s 91.5% accuracy. Conversely, when using LIWC features, XGBoost showed better performance than CNN–BiLSTM.

## 1. Introduction

Suicide represents a significant social issue. Every year, about 700,000 million people take their own lives worldwide, and many more, especially individuals in their twenties and thirties, attempt suicide, according to the World Health Organization (WHO) [1]. Suicide is the second leading cause of death among people aged between 10 and 34 years [2]. Contemplating ending one’s own life is an example of suicidal ideation, which is also commonly referred to as suicidal thoughts. People of all ages may suffer from suicidal ideation for various reasons, including shock, anger, guilt, depression, and anxiety. Long-term depression may lead to suicide if adequate therapy is not sought, despite the fact that the vast majority of individuals who experience suicidal thoughts do not actually attempt to end their own life [3]. Suicidal ideation can be managed with the assistance of healthcare professionals and medications. However, most people with suicidal ideation avoid medical treatments due to the stigma associated with them. Instead, many people choose to communicate their intent to commit suicide on social media. Because mental illness may be diagnosed and treated, the early identification of warning signs or risk factors may be the most effective way of preventing suicide.

Suicidal ideation is a propensity to end one’s life and may vary from depression to a plan to commit suicide [2]. Suicidal ideation is described as a tendency to terminate one’s life. There is considerable debate among researchers about the link between these two categories. Klonsky et al. [4] argued that the most often reported risk factors for suicide (depression, hopelessness, and frustration) were the predictors of suicidal thoughts rather than the shift from suicidal ideation to actual attempt. On the other hand, a person who has suicidal thoughts and a person who has tried suicide may share many common factors, since there are “many variables identified as risk factors for suicidal action”, as stated by Pompili et al. [5]. The WHO member nations collaborated to develop early suicidal ideation detection tools, with the common objective of lowering suicide rates by ten percent by the year 2020 [6].

Sentiment analysis is a rapidly developing technique that can automatically capture users’ feelings [7,8]. Using information available on social media, sentiment analysis can identify early signs of suicidal ideation and prevent attempts at suicide. As a direct consequence of this, machine learning (ML) and natural language processing (NLP) are increasingly used to infer suicidal intent from social media content [8]. Previous studies used ML algorithms to identify suicidal ideation in tweets using small datasets. In [9], depression was identified in a sample of 15,000 tweets using multiple ML models. The authors of the paper [10] increased the performance of machine learning (ML) classifiers by utilizing a dataset of 50,000 tweets that were manually tagged to conduct a binary classification after being acquired from a variety of online and news articles using keywords. An automatic depression detection method was developed in [11], where the authors used ML models to analyze a dataset obtained from the Russian social networking platform Vkontakte. However, because these studies used limited datasets, their models did not achieve high accuracy. The classification accuracy of ML models can be increased by applying relevant annotation rules to large volumes of data and by training deep learning (DL) models [12]. 

The following summarizes the most important contributions of this paper:

- For the purpose of identifying suicidal tendencies, the proposal of a hybrid deep learning model that combines convolutional neural networks with bidirectional long-short term memories;
- Evaluation of how well the suggested deep learning model performs in comparison to the XGBoost machine learning model that serves as a baseline.
- Conducting of two different experiments using text and LIWC-based features to test the performance of the proposed models.
- Analysis of the suicide and non-suicide posts in the dataset and concluding the difference between them using the LIWC tool.

## 2. Background

In recent years, several experiments in many countries examined the potential of using social media to identify individuals with suicidal ideation. Markers of the shift from mental health discourse to suicidal ideation were derived by Choudhury et al. [13] using a statistical method that was based on a score-matching model. The first stage is characterized by anxious thoughts, feelings of helplessness, and sadness. The second stage is characterized by reduced levels of social cohesion and self-esteem. The third stage is characterized by hostility and a plan to commit suicide. Coppersmith et al. [14] investigated changes in users’ behavior and found a significant increase in tweets expressing sentiments of melancholy in the weeks leading up to an attempt at suicide. Moreover, in the weeks following a failed attempt, there was a substantial rise in tweets reflecting rage.

In the last several years, a considerable amount of research also studied the relationship between mental health and language use in order to acquire new insights into how suicidal thoughts may be identified and how they might be prevented. For the aim of this study, linguistic characteristics that are established in the field of psychiatry, such as the LIWC [15], emotion features [16], and suicide notes [17], were used. However, this method employs language-specific strategies that can evaluate only individual posts in isolation and cannot perform well with vast amounts of diverse data.

The use of NLP to analyze social media posts for the study of mental health is gaining increasing popularity. Sentiment analysis is increasingly used on social and mental health–related forum data. For example, Tadesse et al. [18] built a combination model using LDA, LIWCA, and MLP, and achieved 90% accuracy. In [9,10,11], the researchers collected data from Twitter using a method similar to that described here and then employed different ML approaches to categorize suicidal ideas.

As word embedding is becoming increasingly common, DL methods, such as long short-term memory (LSTM) and convolutional neural networks (CNN), are leading to considerable advances in the field of NLP. Because ML methods are subject to several constraints, including dimension explosion, data sparsity, and long processing times, they cannot be used for every application. Traditional machine learning approaches have the potential to benefit greatly through the use of deep learning (DL) approaches, which allows the significant features from input data. Increasing the number of layers in a model is one way to achieve high accuracy. As a consequence, the model will provide a classification that is both more accurate and more trustworthy. It was established in [19,20] that DL models are superior to ML classifiers because they attained a greater level of accuracy in the prediction of suicidal thoughts. Tadesse et al. [21] used a CNN–LSTM model with Word2Vec to predict suicidal thoughts, with an accuracy of 93.8%. This was made possible by the model’s capacity to extract both long-term global dependencies and local semantic information; however, the authors used a limited dataset.

The accurate detection of suicidal tendencies based on the recognition of regular linguistic patterns in social media posts is an important step toward preventing suicide attempts. NLP methods were used with a number of different ML methodologies. By evaluating suicide notes using binary support vector machine (SVM) classifiers, Desmet et al. [22] developed a technique for predicting suicidal thoughts. This approach may be found in their paper. A psychological vocabulary was established by Huang et al. [23] and was generated from a Chinese feeling dictionary (HowNet). The authors constructed a real-time detection system for suicidal thoughts that was applied on Chinese Weibo and utilized SVM to establish a classification system. Researchers Braithwaite et al. [24] identified people who were at danger of committing suicide by using machine learning algorithms. Language framing was shown to be a key component in Sueki et al. [25]’s research on the suicide intent of Japanese Twitter users in their twenties, which demonstrated the importance of detecting suicidal signals in text. For instance, the statement “want to commit suicide” was associated with lifelong suicidal intent more often than the expression “desire to die”. O’Dea et al. [26] used both human codes and automated machine learning classifiers (LR and SVM) to term frequency-inverse document term frequency (TF-IDF) variables in order to identify the amount of fear that was present in postings that were connected to suicide. Researchers Wood et al. [27] found 125 individuals on Twitter and followed their posts before the individuals committed suicide. They were able to detect the gender of the users with 91.9% accuracy by using simple and linear classifiers, and they discovered that 70% of the users had made at least one attempt at suicide. Okhapkina et al. [28] modified information retrieval techniques to detect pernicious informational influences in social networks and compiled a lexicon of terminology associated with suicidal thoughts and behaviors. Moreover, they pioneered the use of TF-IDF matrices and singular vector decompositions for such matrices. Sawhney et al. [29] increased the effectiveness of a random forest classifier in detecting suicidal ideation in tweets. Aladag et al. [30] used logistic regression classification algorithms to detect suicidal content with an accuracy of 80–92%.

The use of neural network models in NLP for detecting suicidal ideation using sophisticated DL architectures can outperform traditional ML systems. Recurrent neural networks (RNNs) can be used effectively in sequences [31]. LSTM can preserve relevant information free from long-range dependencies. Sawhney et al. [32] demonstrated the superiority of C–LSTM-based models to other DL and ML classifiers in detecting suicidal ideation. Ji et al. [33] compared an LSTM classifier with five ML models, demonstrating the applicability of the various techniques. Their study provided one of the primary criteria for detecting suicidal ideation on social media platforms, such as Twitter and Reddit SuicideWatch.

## 3. Materials and Methods

**Figure 1:**
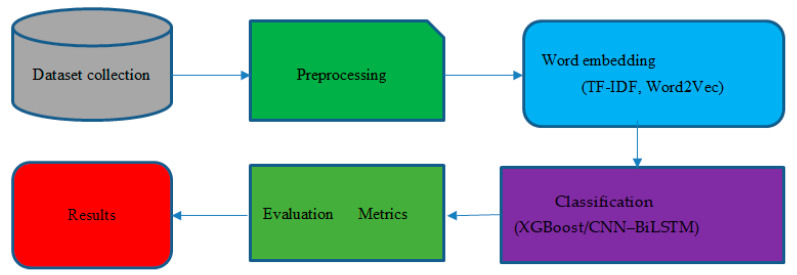
Framework of the proposed suicide ideation detection system.

This section presents the main components of the proposed suicidal ideation detection system (SIDS) framework using linguistics, signs, and activities on SuicideWatch, which is a sub-platform of the Reddit social media news aggregation platform. Figure 1 presents the steps of this framework.

This is the most important stage of our experimental work on online suicide ideation detection.

### 3.1. Dataset

We used a publicly available Reddit dataset downloaded from the Kaggle website. The dataset comprised 232,074 posts to SuicideWatch from 16 December 2008–2 January 2021, including 116,037 suicidal and 116,037 non-suicidal posts. The term “suicide watch” refers to a monitoring procedure intended to prevent suicide attempts. The term typically applies to individuals in jails, hospitals, mental facilities, and army bases. Individuals suspected of displaying suicide warning signals, meaning that they may be at risk of intentional self-harm, are placed under suicide watch.

### 3.2. Preprocessing

This step aims to filter textual posts to eliminate noise before applying feature extraction and embedding techniques, and to produce a word vector for classification. It includes stop word removal, punctuation removal, lowercasing, tokenization, and lemmatization. We employed the Natural Language Toolkit (NLTK) [34] and performed basic tasks to preprocess the dataset.

- Punctuation, emoji, and numerical digit removal: this process removes the characters “?, !, :, ;, ’,” and emoji to make the text easily processable.
- Stop word removal: this process removes words such as “the”, “a”, “an”, and “in”, which have no contribution to the operation of the model.
- Lowercasing: this process lowercases all words.
- Tokenization: this process splits each sentence into its basic parts, such as words, phrases, and other pieces of information.
- Lemmatization: this process combines inflected forms of words into their root form.
- To use a DL neural network technique to distinguish between suicidal and non-suicidal posts, all sequences of texts in the dataset must have equal real-value vectors. To accomplish this task, the post-padding sequence method was used.

### 3.3. Word Embedding

Word embedding is a text representation process widely used for language modeling and feature representation in NLP. It converts each word and sentence of a given text into low-dimensional feature vectors to be analyzed by ML algorithms. In this work, we used TF-IDF [3] and Word2Vec [35] to extract vector representations of words and sentences for suicidal/non-suicidal classification.

#### 3.3.1. TF-IDF

TF-IDF is a representation and feature extraction approach used in text categorization models [36] and is widely employed for understanding natural language and information retrieval. This statistical method is particularly used to measure the importance of a pattern in a text. Its first component, *TF*, identifies the occurrence of specific words to determine the similarity between them, as follows:

(1) $$TF{\left(w\right)}_{d}=\frac{n_{w}\left(d\right)}{\;\left|d\right|}$$

Set *D* points to a set of documents, and d denotes a single document,  d∈ D . Each document is represented as a group of sentences and words w, and $n_{w}\left(d\right)$ is the number of recurrent words w in document d. Therefore, the size of document d is calculated as follows:

(2) $$\left|d\right|=\sum_{w\;\in \;d}n_{w\;}\left(d\right)$$

The frequency at which a word appears in the document is expressed in Equation (2).

*IDF*, the second component of *TF-IDF*, is used to compute the number of documents in a textual corpus in which a specific word appears, as follows:

(3) $$IDF{\left(w\right)}_{d}=1+log\left(\frac{\;\left|D\right|\;}{\left|\;\left\{d\;:\;D|w\;\in d\right\}\right|}\right)\;$$

The *TF-IDF* for word w associated with document d and corpus *D* can be calculated as:

(4) $$\mathrm{TF}-\mathrm{IDF}=TF{\left(w\right)}_{d\;}\times IDF{\left(w\right)}_{D}$$

Generally, *TF-IDF* uses a document-term matrix to generate different text classification systems.

#### 3.3.2. Word2Vec

Word2Vec is another method for obtaining word embeddings—that is, for extracting numeric representations of words—in a given text that is widely used in language modeling and feature learning. This algorithm, developed by Google, has a two-layer neural network structure to extract vector representations and predict the context of a given word in a text. Although it has a limit to handling words that are not in the selected vocabulary size as maximum features, it is nevertheless optimal for NLP tasks [37] since it finds associations between words and sentences. In this work, Word2Vec was used to convert and map each word in the dataset used for training and testing into a 32-dimensional word representation vector.

### 3.4. Classification Models

After obtaining word embeddings for each post content using TF-IDF and Word2Vect, supervised ML algorithms—namely, Extreme Gradient Boosting (XGBoost) and a hybrid CNN–bidirectional LSTM (BiLSTM) DL algorithm—were used for classification. In this study, we compared the performance of XGBoost using TF-IDF word features with that of CNN–BiLSTM using Word2Vec word embeddings.

#### 3.4.1. XGBoost Model

XGBoost is a supervised ML technique widely used in classification and regression tasks. It has a structure similar to that of the gradient decision tree algorithm, which has high-speed performance. Its design aims to use memory and computing resources efficiently. Its implementation involves several characteristics. It applies sparse awareness, automatically addressing missing data values. It uses a block architecture to facilitate parallel tree creation. Repeated training with previously fitted data can improve algorithm performance [38].

#### 3.4.2. CNN–BiLSTM Model

**Figure 2:**
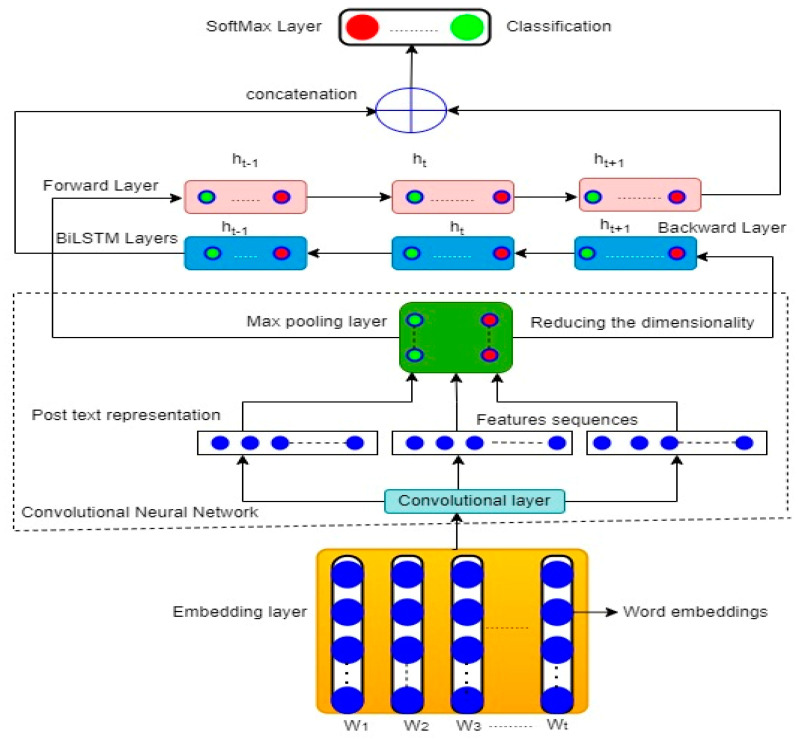
Structure of the CNN–BiLSTM model.

The CNN–BiLSTM model used in this study includes an embedding layer, a convolutional layer, a max pooling layer, bidirectional LSTM layers, and a softmax classification layer. Figure 2 shows the structure of the CNN–BiLSTM model.

1. Embedding layer

This is the first hidden neural network layer. Its structure is based on three parameters of the CNN–BiLISTM architecture: input sequence length, embedding dimension, and maximum features. The input sequence length is the average length of each post in the dataset, which was set to 430 words. Maximum features are the 30,000 most recurring words extracted from the training dataset using Word2Vec (represented in Figure 2 by W1, W2, …, Wn). The embedding dimension adopts the size vector of each word vectorized into sequences of integers, and it was specified as 32-dimensional word vectors. The main task of the embedding layer is to create an input embedding matrix for each word selected from the training, as follows:

(5) $$E(w)=R^{V\times D}$$

where *E*(*w*) is the embedding matrix, *R* is a real number system, *V* is the vocabulary size (maximum features), and *D* is the dimension of the word embedding vector.

2. Convolutional layer

The input data for this layer are a word-embedding matrix that is merged by applying a convolutional operation to construct feature maps [39]. The convolutional layer performs computations on the input-embedding matrix for selected words provided by an embedding layer. It uses filters to pass across the matrix to collect sequence information and reduce the dimensions of the input sequence. It uses four main parameters—number of filters, kernel size, type of padding required, and nonlinear activation function—to produce the feature map to the following layer. The convolutional operation is expressed by

(6) $$y_{j}^{l}=\sigma \;\left(\;\sum_{i=1}^{N_{i-1}}conv(w_{i,j}^{l},\;x_{i}^{l-1})\right)\;+b_{j}^{l}$$

where $N_{i-1}$ represents the number of the feature map, $y_{j}^{l}$ is the feature map of the word embeddings in the social media posts, $\;w_{i,j}^{l}$ denotes the convolutional kernel, $b_{j}^{l}$ is the bias of the feature map, and σ is a rectified linear unit (ReLU) activation function.

3. Max pooling layer

In this layer, the feature map is passed using the convolutional kernel. It performs a pooling operation on the feature map matrix by calculating the maximum value from a pooling window and uses it to reduce the dimensionality of the downsampled feature map of an input sequence to enhance the model’s classification performance. The equation

(7) $$Q_{i}=Max(P_{j}^{1},\;P_{j}^{2},P_{j}^{3},\ldots .,P_{j}^{t})$$

where $Q_{i}$ indicates the output from the max pool, and $P_{j}^{t}$ is the feature map before maximization.

4. BiLSTM layers

LSTM, a type of RNN, is used in various artificial intelligence and DL tasks, including NLP, image processing, sequence mining, and text mining [40]. It is capable of learning long-term dependencies, which allows it to retain information for long periods. The memory cells employed in LSTM can ultimately transfer the results of prior data features to the output. However, feature learning occurs only in a forward direction, thus ignoring backward construction and reducing the performance of the ML system. To tackle this problem, BiLSTM has two hidden layers in opposite directions connected to a single output. Thus, the input training data are processed in both forward and backward directions. The structure of the hidden layers includes four gates, which determine the amount of past sequence information that should be ignored and the amount of context that should be carried forward. This makes BiLSTM ideal for identifying suicidal content in social media posts. The four gates are input $\;i_{t}$ , forget $\;f_{t}$ , cell state $c_{t}$ , and output gate $\;o_{t}$ . The equations for these gates are as follows [41,42,43,44,45,46]:

(8) $$\;i_{t}=\sigma \left(W_{ix}x_{t}+W_{ih}h_{t-1}+b_{i}\right)$$

(9) $$\;f_{t}=\sigma \left(W_{fx}x_{t}+W_{fh}h_{t-1}+b_{f}\right)$$

(10) $$\;o_{t}=\sigma \left(W_{ox}x_{t}+W_{oh}h_{t-1}+b_{o}\right)\;$$

(11) $$c_{t}=f_{t}c_{t-1}+i_{t}\times \tanh\left(\;W_{cx}x_{t}+W_{ch}h_{t-1}+b_{c}\right)$$

(12) $$\vec{\;h_{t}}=o_{t}\times tanh\left(c_{t}\right)$$

(13) $$\overset{\leftarrow }{\;h_{t}}=o_{t}\times tanh\left(c_{t}\right)$$

(14) $$\tanh\left(x\right)=\frac{\;1-e^{2x}}{\;1-e^{2x}}$$

(15) $$\;H_{t}=\left(\vec{\;h_{t}}\;∶\overset{\leftarrow }{\;h_{t}}\right)$$

where sig and tanh represent the sigmoid and tangent activation functions, respectively, *x* represents the input sequences, *W* and *b* are the weight and bias, respectively, $C_{t}\;$ indicates the cell state, $\;h_{t}$ denotes the output of the LSTM cell, and $\;H_{t}$ is the output of the bidirectional concatenation of the $\vec{\;h_{t}}$ forward and $\overset{\leftarrow }{\;h_{t}}$ backward LSTM layers at time *t*.

5. Softmax layer

This is an output layer [42] that we used to estimate the probability of a post being suicidal or non-suicidal. To avoid vanishing problems, the softmax function uses a text feature vector acquired as a consequence of the LSTM layers and divides it into dataset classes. The function is expressed as

(16) $$\sigma \left(z\right)=\frac{e^{z_{i}}}{\begin{array}{c} \;\sum_{j=1}^{K}\;e^{z_{j}}\; \end{array}}$$

where *z* denotes the values of the neurons placed in the output layer, and *e* is an exponential that acts as a nonlinear function. Table 1 presents the parameters used in the DL model.

### 3.5. Evaluation Metrics

To evaluate the performance of the CNN–BiLSTM and XGBoost models in classifying post content as suicidal or non-suicidal, we used common evaluation metrics with a focus on the number of false-positive and false-negative classifications obtained from the confusion matrix presented. The performance metrics used were *Accuracy*, *Precision*, *Recall*, *Specificity*, and *F*1-score, which were calculated as follows:

(17) $$Accuracy=\frac{TP+TN}{FP+FN+TP+TN}\times 100$$

(18) $$Precision=\frac{TP}{TP+FP}\times 100$$

(19) $$Recall=\frac{TP}{TP+FN}\times 100$$

(20) $$Specificity=\frac{TN}{TN+FP}\times 100$$

(21) $$F1–score=2\times \frac{precision\times sensitivity}{precision+sensitivity}\times 100$$

## 4. Experimental Results

This section presents the empirical results obtained from the experiments conducted to detect suicidal ideation in social media posts using textual and Linguistic and Word Count (LIWC-22) [16] features. In the first experiment, based on the learning of word embeddings extracted from post content using TF-IDF and Word2Vec, we used supervised ML (XGBoost) and hybrid DL (CNN–BiLSTM) to create an SIDS that can be used to classify social media posts as suicidal or non-suicidal. In the second experiment, we also used numerical features extracted using LIWC-22.

### 4.1. Data Splitting

In this step, we split the entire dataset into training, testing, and validation subsets to analyze the performance of the proposed SIDS. Table 2 shows the results of the splitting process.

### 4.2. Classification Results

**Figure 3:**
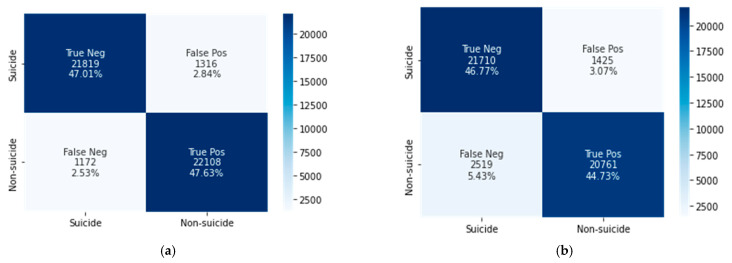
Confusion matrices of (**a**) CNN–BiLSTM and (**b**) XGBoost using textual features.

**Figure 4:**
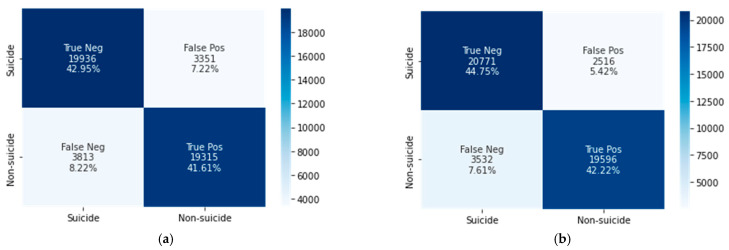
Confusion matrices of (**a**) CNN–BiLSTM and (**b**) XGBoost using LIWC features.

This subsection presents the classification results of the CNN–BiLSTM and XGBoost models, which were trained, validated, and tested using the textual and psychometric linguistic (LIWC) features extracted from Reddit posts (Table 2). Figure 3 and Figure 4 show the confusion matrices of the CNN–BiLSTM and XGBoost models obtained from the textual and LIWC features. Table 3 and Table 4 present the evaluation of the classification results using textual and LIWC features based on the standard metrics of accuracy, recall, precision, specificity, and F1-scores computed from the confusion matrices.

True positive (TP) and true negative (TN) represent the total numbers of samples correctly classified as non-suicidal and suicidal posts, respectively. False positive (FP) and false negative (FN) represent the numbers of samples incorrectly classified as non-suicidal and suicidal posts, respectively.

The CNN–BiLSTM model achieved 95% accuracy using textual features and 84.5% accuracy using LIWC features, whereas XGBoost achieved accuracies of 91.5% and 86.9%, respectively. We can conclude that textual data processing using the DL algorithm is more time-consuming but shows better performance than the ML XGBoost technique. Conversely, in LIWC feature processing, XGBoost achieves slightly higher classification accuracy.

### 4.3. Statistical Analysis of Suicidal and Non-Suicidal Posts

**Figure 5:**
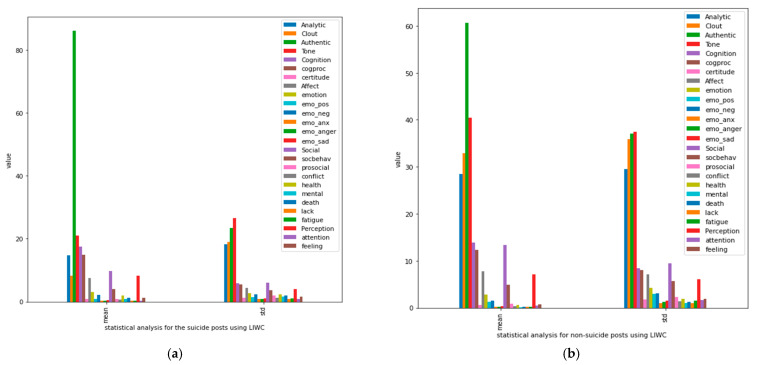
Graphical representation of the statistical analysis of (**a**) non-suicidal and (**b**) suicidal posts determined based on LIWC features.

In this subsection, we present a statistical analysis of distinguishing between suicidal and non-suicidal posts based on psychometric LIWC features. Figure 5 presents the results based on means and standard deviations.

As shown in Figure 5, suicidal posts had higher values of authenticity, anxiety, mentality, depression, negativity, and sentimental despondency than non-suicidal posts. Thus, we can conclude that Reddit users at risk of committing suicide exhibit psychological and mental health problems. Furthermore, these users scored lower on attention, mind-thinking, perception, sociality, and cognitive processes than those who had no intention of committing suicide.

### 4.4. Performance Plots

To further analyze the experimental results, we drew learning curves to visualize the performance of the CNN–BiLSTM model in terms of training and validation accuracy in each epoch.

**Figure 6:**
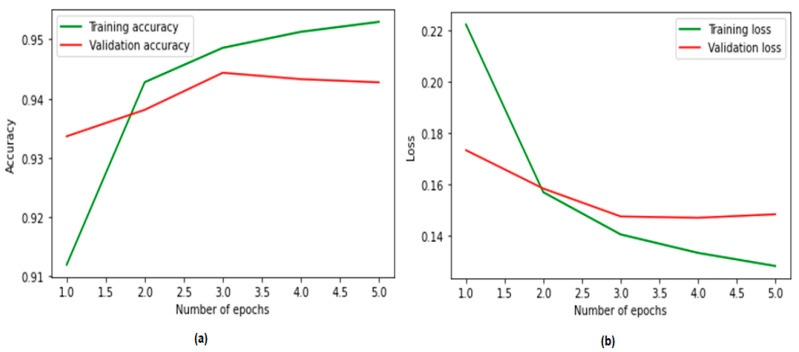
Training and validation (**a**) accuracy and (**b**) loss using textual features.

Figure 6 depicts the validation performance of the suggested model for detecting suicidal ideation. These tests were conducted to evaluate the effectiveness of the proposed model. After beginning with an accuracy of 94%, the CNN–BiLSTM model achieved a validation accuracy of 95% during the course of an operating period consisting of 20 epochs. With the use of cross-entropy measurements, the validation loss was reduced to a value as low as 0.18, which is a considerable improvement when compared with the initial value of 0.14.

**Figure 7:**
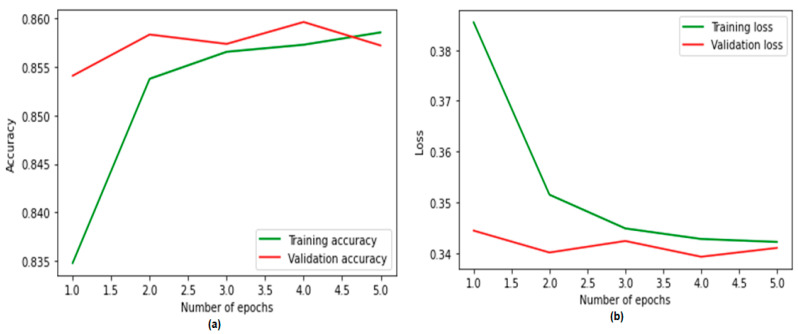
Training and validation (**a**) accuracy and (**b**) loss using LIWC features.

In terms of precision, the degrees of accuracy of the proposed system are shown in Figure 7. On the y-axis is shown the percentage of data that were successfully categorized. The efficiency of the validation system serves as the yardstick by which to measure the accuracy of the training system. We were able to notice a disruption in the process of optimizing the system, which resulted in an improvement in accuracy to 20 epochs, which is outstanding. During the phase of validation, the performance of the CNN–BiLSTM model increased to 83%, up to 84%. A categorical cross entropy function was used in order to quantify the training losses that are associated with the suggested system. After 20 epochs, the validation losses decreased to 0.33 from 0.34.

### 4.5. Word Cloud

**Figure 8:**
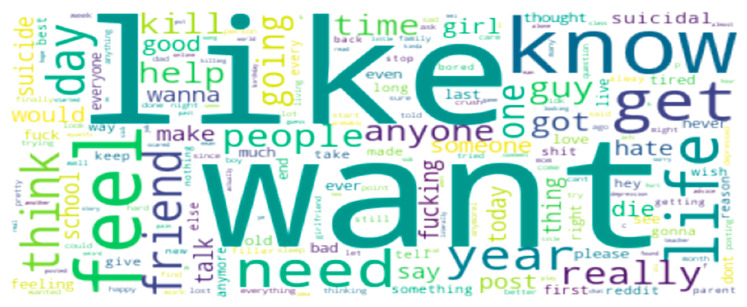
Word cloud based on the dataset.

Word clouds are widely used in NLP to visualize the most important and recurrent words in a textual corpus. Here, we used a word cloud to visualize the most repeated words in the Reddit dataset, shown in Figure 8.

## 5. Discussion

The identification of suicidal ideation at an early stage is a vital and efficient strategy for the prevention of suicide. The vast majority of research pertaining to this topic was conducted by psychologists through the use of statistical analysis. On the other hand, the vast majority of research conducted by computer scientists was conducted through the use of feature engineering-based machine learning and deep learning representation learning. It will be much easier for medical professionals to identify potentially suicidal individuals and save many lives if early suicidal thoughts can be detected on microblogging sites such as Twitter. Both the deep learning and machine learning techniques have the potential to provide novel prospects for enhancing the early identification of suicidal thoughts and the subsequent early prevention of suicide.

In this work, we aimed to evaluate and assess several ML and DL models for determining whether or not user tweets include indicators indicating suicide thoughts and for determining which models performed the best. The primary objective of the research was to determine which model is most effective in recognizing suicidal ideations among Twitter users and able to do so with a high degree of precision.

This section presents a comparative analysis of the proposed models’ performance for suicide ideation detection using text-based features with previous deep learning and baseline machine learning models using the accuracy metric. According to the literature review, no previous research conducted experiments on the same number of dataset samples as used in this research work. However, to compare the performance of our proposed deep learning model with existing methods based on different distributions of the existing Reddit dataset samples, we selected several research articles which used the same data source for the comparison task. Table 5 shows a comparative analysis using the accuracy metric.

## 6. Conclusions

In this study, we developed and evaluated a suicide ideation detection system using ML and hybrid DL techniques to evaluate Reddit users’ psychological states. Our experimental results showed that the CNN–BiLSTM model outperformed the XGBoost model, achieving 95% accuracy in detecting suicidal ideation using textual features, compared with the latter’s 91.5% accuracy. Our analysis using the LIWC lexicon reveals that suicidal posts score higher on authenticity, anxiety, mentality, depression, negativity, and sentimental despondency and lower on attention, mind-thinking, perception, sociality, and cognitive processes than non-suicidal posts. This suggests that Reddit users at risk of committing suicide may exhibit psychological and mental health problems. Nowadays, individuals are more sensitive than ever before, which causes major losses to their families, friends, and others in their immediate environments. By detecting suicidal intent in users’ posts, the proposed system may help identify individuals who require medical treatment and reduce suicide rates.

## Figures and Tables

**Table 1:** Parameters and their values used in the CNN–BiLSTM model.

Parameter	Value
Input sequence length	430
Embedding dimension	32
Vocabulary size	30,000
Number of filters	100
LSTM units	100
Dropout	0.3
Batch size	64
Number of epochs	5
Activation function	ReLU
Optimizers	RMSprop (textual features) + Adam (LIWC features)

**Table 2:** Dataset splitting.

Dataset	Total Samples	Training (70%)	Validation (10%)	Testing (20%)
Reddit (SuicideWatch)	232,074	162,452	23,207	46,415

**Table 3:** Test results using textual features.

Algorithm	Precision (%)	Recall (%)	Specificity (%)	F-score (%)	Accuracy (%)
CNN–BiLSTM	94.3	94.9	94.3	95	95
XGBoost	93.5	89.1	93.8	91.3	91.5

**Table 4:** Test results using LIWC-based features.

Algorithm	Precision (%)	Recall (%)	Specificity (%)	F-score (%)	Accuracy (%)
CNN–BiLSTM	85.2	83.5	85.6	84.3	84.5
XGBoost	88.6	84.7	89.1	86.6	86.9

**Table 5:** Comparative analysis of the performance of proposed model with other existing methods.

Paper Id	Dataset Distribution	Word Representation Approach	Model	Results
**Ref** [21]	3549 suicide indicative posts and 3652 non-suicidal	Word2Vec	LSTM-CNN	93 % accuracy
**Ref** [20]	3549 suicide posts and 3652 non-suicidal	Word2Vec	LSTM	92% accuracy
**Ref** [47]	785 suicide posts and 785 non-suicidal	TF-IDF	SVM	92% accuracy
**Proposed model**	116,037 suicide and 116,037 non-suicide posts	Word2Vec	CNN–BiLSTM	95% accuracy

## Data Availability

The data presented in this study are available here: https://www.kaggle.com/datasets/nikhileswarkomati/suicide-watch, accessed on 22 July 2022.
